# Active-Scanned Protons and Carbon Ions in Cancer Treatment of Patients With Cardiac Implantable Electronic Devices: Experience of a Single Institution

**DOI:** 10.3389/fonc.2019.00798

**Published:** 2019-08-22

**Authors:** Katharina Seidensaal, Semi Ben Harrabi, Eberhard Scholz, Malte Ellerbrock, Thomas Haberer, Fabian Weykamp, Matthias Mattke, Stefan E. Welte, Klaus Herfarth, Jürgen Debus, Matthias Uhl

**Affiliations:** ^1^Department of Radiation Oncology, Heidelberg University Hospital, Heidelberg, Germany; ^2^Heidelberg Institute of Radiation Oncology (HIRO), Heidelberg, Germany; ^3^National Center for Tumor Diseases (NCT), Heidelberg, Germany; ^4^Department of Radiation Oncology, Heidelberg Ion-Beam Therapy Center (HIT), Heidelberg University Hospital, Heidelberg, Germany; ^5^Clinical Cooperation Unit Radiation Oncology, German Cancer Research Center (DKFZ), Heidelberg, Germany; ^6^Heidelberg Center for Heart Rhythm Disorders (HCR), Heidelberg, Germany; ^7^Department of Cardiology, Heidelberg University Hospital, Heidelberg, Germany; ^8^DZHK (German Centre for Cardiovascular Research), Partner Site Heidelberg/Mannheim, Heidelberg, Germany; ^9^German Cancer Consortium (DKTK), Partner Site Heidelberg, Heidelberg, Germany

**Keywords:** particle therapy, beam scanning, cardiac implantable electronic device, CIED malfunction, carbon ion radiotherapy, proton radiotherapy

## Abstract

**Background:** Ionizing radiation was shown to be able to influence the function of cardiac implantable electronic devices (CIED's) leading to malfunctions with potentially severe consequences. Those effects presumably correlate with beam energy and neutron production. Thus, particle facilities are commonly cautious to treat patients with CIED's with particles, but substantial evidence is lacking.

**Methods and Materials:** In total 31 patients were investigated, who have been treated at the Heidelberg Ion-Beam Therapy Center (HIT) from September 2012 to February 2019 with protons and carbon ions in active-scanning technique. All CIED's were checked after every single irradiation by the department of cardiology. The minimum distance between the CIED and the planning target volume (PTV), the 10% isodose and the single beam in Beam's Eye View (BEV) was analyzed for 12 patients.

**Results:** In total, 31 patients received 32 courses of radiotherapy (RT). Twenty-two received treatment with carbon ion beam and ten with proton beam. The cumulative number of fractions was 582, the cumulative number of documented controls after RT was 504 (87%). Three patients had an implantable cardioverter-defibrillator (ICD) and 28 patients had a pacemaker at the time of treatment. Seven patients had a heart rate of ≤30/min. The majority of patients (69%) were treated for tumors of the head and neck. The median minimum distance between CIED and PTV, 10% isodose and the single beam on BEV was 13.4, 11.6, and 8.3 cm, respectively. There were no registered events associated with the treatment in this evaluation.

**Conclusion:** Treatment of CIED-patients with protons and carbon ions applied with active raster scanning technique was safe without any incidents in our single center experience. Monitoring after almost every fraction provided systematic and extensive data. Further investigations are necessary in order to form reliable guidelines, which should consider different modes of beam application, as active scanning supposedly provides a greater level of safety from malfunctions for patients with CIED undergoing particle irradiation.

## Introduction

Population aging and broader indications for the implantation of cardiac implantable devices are responsible for their continuously increasing use. From 1993 to 2008 an increase of 96% was reported in the United States (1). Furthermore, there is a growing burden of comorbidities resulting in continuously rising numbers of oncologic patients with implanted CIED's. Although most medical treatments pose little risk for device function, ionizing radiation has the potential to harm CIED's temporarily or permanently causing malfunctions with potential severe consequences. Most pacemakers (PM) are implanted in patients with or at risk of inappropriate bradycardia. Cardiac resynchronization devices synchronize and coordinate the contractions of the heart and are used in patients with heart failure. ICD's are more sophisticated and have the ability to monitor the heart rate and deliver shocks when e.g., the heart rate exceeds certain criteria. They are considered more sensitive to malfunction by RT compared to PM's (2–4). Generally, there is consent that eletromagnetic interference (EMI) does not pose increased danger to contemporary CIED's as they are well-protected and modern linear accelerators are sufficiently shielded. The mechanisms of CIED malfunction base on the effects of direct or scattered ionizing radiation. Basically transient effects, manifesting only during irradiation, temporary events as reverting to back-up mode, that can be solved by reprogramming as well as permanent malfunctions can be observed. Others discriminate between soft and had errors, meaning alterations of the software or damage to the hardware (5). Patient's characteristics, especially PM dependency, have to be taken into account when considering the risks of CIED failures. In pacing dependent patients loss of stimulation can cause symptomatic bradycardia. On the other hand loss of stimulation control can lead to ventricular tachycardia “runway pacemaker.” CIED malfunctions occur at higher energies, which is attributed to secondary neutron formation. A significant fraction of radiation in particle therapy consists of secondary neutrons, which lead to a general caution in the treatment of patient with CIED's with particles (2, 3). The recommendations on the management of patients with CIED are heterogeneous and most refer exclusively to photon therapy. The current international guideline of the American Association of Physicists in Medicine (AAPM) has been released in 1994 and not been updated ever since, considering the described RT techniques it can be regarded as outdated (6). Hurkmans et al. (3) published a widely accepted guideline in 2012 which however does not include particle therapy. The current German guideline considers particle therapy in the treatment of CIED patients of great concern and offers no safe strategies. However, these remarks are based on a limited number of case reports, one single retrospective study and one experimental setup (7). Due to the excellent dose distribution of particles the number of especially proton facilities is increasing worldwide. Thus, clinical data and reliable guidelines for particle therapy of CIED patients have been anticipated for years. This study demonstrates our experience at particle radiotherapy of CIED patients and contributes to future risk assessment strategies in the management of patients with CIED's undergoing radiotherapy with protons or carbon ions.

## Materials and Methods

### Collected Data and Participants

The Ethics committee of the University of Heidelberg approved the study. Thirty-one consecutive patients who had a CIED and were treated with protons (*n* = 10) or carbon ions (*n* = 22) at Heidelberg Ion-Beam Therapy Center (HIT) from September 2012 to February 2019 were included in this retrospective study. The median prescribed dose was 51 Gy (RBE, relative biological effectiveness) [range 10 Gy (RBE) to 66 Gy (RBE)], 90% of patient had a PM and 10% an ICD. Information on particle type, prescribed radiation dose and fractionation including details of the treatment plan, diagnosis, tumor site, type of CIED, manufacturer of the device, pacemaker-dependence and information generated by the device was collected (Tables 1–3). When patients received combined treatment with a carbon ion or proton boost and a photon primary plan (*n* = 6), the boost plan only was considered for the cumulative number of controlled fractions.

**Table 1:** Baseline characteristics of patients.

	***n***	**Range or %**
Median age at treatment	72	43–89
Gender		
Female	12	39
Male	19	61
Anatomical region (per treatment)		
Head, skull base, head, and neck	22	69
Abdomen and pelvis	6	19
Thorax	4	12
Diagnosis (per patient)		
Head and neck cancer (e.g., adenoidsystic,mycoepidermoid, and squamous cell carcinoma)	10	
Brain tumor (e.g., meningioma and glioma)	8	
Sarcoma (e.g., chondrosarcoma, osteosarcoma), chordoma	7	
Pancreatic cancer	2	
Prostate cancer	2	
Lymphoma	1	
Bronchial cancer	1	

**Table 2:** Treatment characteristics.

		**Range or %**
Median total dose	51 Gy (RBE)	10–66 Gy (RBE)
Median number of fractions per treatment	17.5	5–33
Cumulative number of fractions	582	
Cumulative number of documented controls after single fraction	504	87
Cumulative number of documented controls before beginning of RT (per treatment)	20	63
Particle		
Carbon ion	22	69
Proton	10	31
Single dose		
Carbon ion	3 Gy (RBE)	
Proton	2 Gy (RBE)/1.8 Gy (RBE)	
Bimodal treatment (particle boost plus photon main plan)	6	19
Median number of beams^*^	2	1–4
Median CTV volume (ccm)^*^	107.56	5.88–1956.10
Approx. median treatment time^*^ (min)	6	2–19
Re-irradiation	9	28

* *Refers to the main plan when volume reduction was performed for some fractions of the particle irradiation*.

**Table 3:** CIED characteristics.

	***n***	**%**
CIED type		
ICD	3	10
PM	28	90
PM dependence (per patient)		
Dependent (≤30 bpm)	7	19
Not dependent (>30 bpm)	22	80.4
Missing	2	0.6
Manufacturer (per patient)		
Medtronic	19	61
St. Jude Medical	4	13
Biotronik	4	13
Boston Scientific	3	10
ELA Medical	1	3

In the planning computed tomography (CT) of 12 patients, which received 13 treatments, the CIED was encompassed completely. We analyzed the minimum distance between CIED and the planning target volume (PTV) as well as the distance to the 10% isodose. The minimum distance of the CIED and the single beam was estimated on artificially reconstructed Beam's Eye View (BEV) for 32 beams of 16 plans (1–3 beams per plan) in total. BEV delivers two-dimensional summation radiographs (DRR) digitally reconstructed in the direction of the single beam. Thus, we considered the arrangement of the beam direction in relation to the CIED.

### Internal Guidelines and CIED Evaluation

The decision to treat a patient with particles was not based on tumor localization or pacing dependency, but on the diagnosis and stage of disease alone. All devices were restricted to doses of 2 Gy (RBE) or less and were not located within the treatment field or the proximal beam direction. Relocation of the device is generally considered, when it is not possible to meet these preconditions, which was not the case in this study cohort. Based on our experience, we did not use asynchronous stimulation of pace makers or deactivation of antitachycardia treatment of ICD's through reprogramming or magnet placement. Furthermore, we did not perform continuous monitoring by electrocardiography (EC) or pulse oximetry (Sp0_2_).

For the purpose of this retrospective study, we defined pacing dependence as a heart rate of ≤ 30 beats per minute (BPM). Independent of risk considerations, all patients were seen by a specialist at the department of Cardiac Electrophysiology of the Heidelberg University Hospital after every single fraction. Devices were interrogated for resets, battery status as well as pacing and sensing thresholds with the possibility of reacting to setting alerts and reprogramming in case of malfunctions. We analyzed the available documented controls (87% of all fractions).

### Treatment Facility and Beam Delivery

All patients were treated at Heidelberg Ion-Beam Therapy Center (HIT). Treatment with protons and carbon ions was performed exclusively with active raster scanning as previously published (8, 9).

Cardiac resuscitation equipment was available at the facility at all times. Furthermore, a hospital resuscitation team and access to an intensive care unit was available in case of emergency. Every patient was monitored for clinical changes throughout each fraction with an in-room video, audio and motion system.

## Results

In the observed cohort the cumulative number of fractions was 582, the cumulative number of reported controls of the CIEDs was 504 (87%). Three patients had an ICD, 28 a pacemaker. Seven patients had a heart rate of ≤ 30 BPM. The majority of patients (69%) were treated for tumors of the head and neck. One patient received two consecutive treatments for myoepithelial carcinoma of the submandibular gland, the timeframe between the end of the first and beginning of the second treatment (re-irradiation for recurrence) was 14 months.

**Figure 1:**
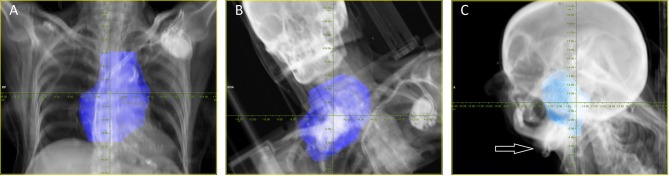
Examples for BEV proton and carbon ion plans. **(A)** Eighty-eight year old patient with diffuse large B-cell lymphoma, treatment was performed with protons up to 40 Gy (RBE) in 20 fractions (one of three beams in total). **(B)** Sixty-seven year old patient with a recurrent chordrosarcoma grade 1 of the upper thorax, treatment was performed with carbon ions up to 60 Gy (RBE) in 20 fractions (one of two beams in total). **(C)** Fifty-eight year old patient with nasopharyngeal cancer, one of two beams aims in the direction of the pace maker (white arrow), re-irradiation was performed with carbon ions up to 51 Gy(RBE) in 17 fractions.

The median minimum distance between CIED and PTV was 13.4 cm (range 4.1–17.9 cm), and the median minimum distance between CIED and the 10% isodose was 11.6 cm (range 2.4–17.1 cm). In two plans with targets in the head and neck the CIED was located in beam direction of one beam behind the target. The distance between beam and CIED on BEV was 0 cm and 0.5 cm. The median distance between the single beam and CIED measured in the direction of the beam was 8.3 cm (range 0–18 cm) (Figure 1).

There were no registered events such as set-ups or other changes of parameters in this reported control. One patient had an enhanced impedance of the device leads, which fluctuated during treatment, however, no intervention was required and the effect was not attributed to radiotherapy, as it was present before treatment initiation.

Additionally, also in the photon part of the combined particle and photon treatments, which were performed and controlled at the same conditions after every single fraction, no malfunctions were observed.

## Discussion

To our knowledge this is the first cohort reporting management of CIED patients undergoing carbon ion radiotherapy and one of the largest cohorts reported on patients with CIED undergoing proton radiotherapy. According to our experience the active scanning beam application mode can be applied with a high grade of safety compared to passive scattering where device malfunction rates of ~20 to 28.6% have been reported previously by others under similar treatment set-up conditions [with pulse generators outside the treatment fields in all cases and restriction to 2 Gy(RBE) and below by Gomez et al. (10)] (10–12).

### Influence of Secondary Neutrons on CIED Function

Several *in-vitro* and *in-vivo* studies have been published on device malfunctions in photon beam therapy. Based on clinical experience it is generally agreed that photon energy (≥15 mega electron volt, MeV) is one of the major risk factors, as several malfunctions were observed for energies of 15–18 MeV, whereas 6–10 MeV seems to bear low risk for resets or other types of malfunction. This observation is explained by a higher rate of neutron production at higher energies, as neutrons are considered to be able to interfere with the electronics of the devices causing mainly device resets. In the largest multicenter cohort (*n* = 560) reported from Denmark by Zaremba et al. (2), the rate for malfunctions in photon treated patients accounts 2.5% for PMs and 6.8% for ICDs. The authors identified location of the tumor below the diaphragm and beam energy as the main factors associated with device malfunction. However, tumor localization was not confirmed as an independent risk factor after adjustment for beam energy.

Raitt et al. (13) reported in 1994 a malfunction of a pacemaker called runway pacemaker in a case where an estimated dose of 0.9 Gy was applied to the device during fast neutron irradiation and demonstrated the sensitivity of integrated circuits. Although neutron irradiation is nowadays obsolete due to an increased rate of late morbidity, parallels can be drawn to secondary scattered neutrons from particle irradiation.

In an experimental setup Hashimoto et al. (5) placed four ICDs outside a 10 × 10 cm proton field (0.3 cm laterally and 3 cm distally). The cumulative in-field dose was 107 Gy in 10 fractions. The rate of power resets (changes to safety back-up mode) was 1 per 50 Gy. In total 29 soft errors occurred in all devices with no permanent errors. Here also the method of beam application was passive scattering and the effects were contributed to secondary scattered neutrons of the estimated dose 1.3–8.9 mSv/Gy (5).

### Influence of Beam Delivery Methods on the Rate of Neutron Production

The rate of production of neutrons in particle therapy depends on the type of beam application mode. Passive scattering, which was the first available technique, yields higher rates of out-of-field scattered neutrons. Here the beam is spread by placing scattering materials in its path and the conformation of dose to target volume is achieved by collimators and compensators. The interaction of the beam with these and other beam line elements leads to neutron production (14).

In an experimental set-up a dose advantage of factor of at least 10 was shown for spot scanning compared to passive scattering techniques. The calculated out-of-field neutron dose for large and medium targets was approximately 0.004 and 0.002 Sv per treatment Gy of protons, respectively (15). Others report this difference to be even higher (16), especially in the out-of-field entrance region by a factor of 30–45, decreasing then with depth (17).

In pencil beam scanning the beam is deflected and steered by magnets, beam modifying components are in many cases not necessary. Compared to passive scattering all beam modifiers (ripple filters, which are always used for carbon ions, range shifters) are located rather close to the patient, reducing the scattering effect for the out-of-field area. With active beam application the high dose area can better be adapted to the shape of the target volume which leads to a significant reduction of dose absorbed by the surrounding healthy tissue. Here, the majority of neutron production occurs within the patient's body. By those differences in modes of beam application, the production of neutrons and the integral dose of the patient are lower for active scanning. When comparing carbon ions with protons, it is worth noting that carbon ions yield a higher neutron rate than protons as a result of a process called nuclear fragmentation, which occurs with all ions heavier than proton. In nuclear reactions lighter ions are created, which continue their path with approximately the same velocity. This process contributes not only lighter ions but also enhances neutron production. Nonetheless, due to a higher linear energy transfer (LET) less particles are necessary to gain the same biologically effective dose and thus the net neutron production is similar (18–20). Furthermore, the penumbra of carbon ions is smaller by the factor of approximately 3 than the penumbra of protons, resulting in less irradiated out-of-field healthy tissue (21).

### Clinical Data on CIED Function in Particle Irradiation

Clinical data highlight the difference in treatment modes. Gomez et al. (10) reported in 2013 of 42 patients which received proton beam therapy (PBT) and reported six CIED malfunctions in five patients, five malfunctions were radiation dependent resets and one elective replacement indicator (ERI) that was not influenced by treatment. The four patients who had resets had passive scattering therapy to the thorax, one patient experienced two subsequent malfunctions. The defects were detected by observation and corrected without any clinical incidents. Both beam application types were used with a higher prevalence of passive scattering in 76%, compared to active scanning in 24% of cases. The authors conclude that quantitative thresholds for distance and dose cannot be derived from the data, but the risk of malfunctions is minimal for fields farther than 30 cm from the CIED. They advise to avoid thoracic PBT for pacing-dependent patients (10). In our cohort only four patients received radiotherapy to the thorax, two were treated by carbon ion and two by proton radiotherapy. Thus, we can neither confirm nor refute this finding. Still it is worth noting that in our cohort even at a minimum distance of 4 cm between PTV and CIED treatment was possible without triggering malfunction (Figure 1A).

Oshiro et al. (11) published a phantom study as well as patient data in 2008. First, they confirmed safety by a phantom study, with pacing leads being placed within two acrylic phantoms in the treatment field and a pacemaker behind the two phantoms outside the treatment field. Although the set-up showed no relevant influence of neutron scatter, when treatment was performed two of eight patients with abdominal radiotherapy with passive scattering showed changes in the heart rate due to resets to safety back-up mode. The patients remained asymptomatic (11).

Ueyama et al. (12) reported two cases of resets to VVI backup mode in a series of seven patients treated by passive scattered protons. One patient received thoracic radiotherapy for bronchial cancer and one abdominal for pancreatic cancer and in both cases resets were not detected by ECG and were not clinically apparent otherwise (12).

## Conclusion

Despite the retrospective character and the small number of patients, the study provides important evidence that particle treatment of patients with CIED's can be safely performed with active scanning of protons and carbon ions. Considering these findings, one has to point out that future guidelines should discriminate not only between photon and particle treatment but also between the modes of particle beam application, namely passive scattering and active scanning, with the latter possibly offering a superior level of safety for CIED patients.

## Data Availability

The raw data supporting the conclusions of this manuscript will be made available by the authors, without undue reservation, to any qualified researcher.

## Ethics Statement

This study was performed following institutional guidelines and the Declaration of Helsinki of 1975 in its most recent version. This study was approved by the Independent local Ethics Committee Heidelberg (Ref. Nr. S-131/2019).

## Author Contributions

KS, JD, and MU contributed conception and design of the study. SH, ES, FW, MM, SW, and KH provided the data. KS, TH, ME, ES, and MU analyzed the data. KS, SH, MU, ME, and JD wrote the manuscript. All authors contributed to manuscript revision, read, and approved the submitted version.

### Conflict of Interest Statement

The authors declare that the research was conducted in the absence of any commercial or financial relationships that could be construed as a potential conflict of interest.
